# Calreticulin is a Critical Cell Survival Factor in Malignant Neoplasms

**DOI:** 10.1371/journal.pbio.3000402

**Published:** 2019-09-30

**Authors:** Arum Han, Chen Li, Tara Zahed, Michael Wong, Ian Smith, Karl Hoedel, Douglas Green, Alexander D. Boiko

**Affiliations:** 1 Sue and Bill Gross Stem Cell Research Center, Department of Molecular Biology and Biochemistry, University of California–Irvine, Irvine, California, United States of America; 2 Center for Autism Research and Translation, University of California–Irvine, Irvine, California, United States of America; 3 Department of Immunology, St. Jude Children's Research Hospital, Memphis, Tennessee, United States of America; Buck Institute for Research on Aging, UNITED STATES

## Abstract

Calreticulin (CRT) is a high-capacity Ca^2+^ protein whose expression is up-regulated during cellular transformation and is associated with disease progression in multiple types of malignancies. At the same time, CRT has been characterized as an important stress-response protein capable of inducing immunogenic cell death (ICD) when translocated to the cell surface. It remains unclear why CRT expression is preserved by malignant cells during the course of transformation despite its immunogenic properties. In this study, we identify a novel, critical function of CRT as a cell survival factor in multiple types of human solid-tissue malignancies. CRT knockdown activates p53, which mediates cell-death response independent of executioner caspase activity and accompanied full-length poly ADP ribose polymerase (PARP) cleavage. Mechanistically, we show that down-regulation of CRT results in mitochondrial Ca^2+^ overload and induction of mitochondria permeability transition pore (mPTP)-dependent cell death, which can be significantly rescued by the mPTP inhibitor, Cyclosporin A (CsA). The clinical importance of CRT expression was revealed in the analysis of the large cohort of cancer patients (*N* = 2,058) to demonstrate that high levels of CRT inversely correlates with patient survival. Our study identifies intracellular CRT as an important therapeutic target for tumors whose survival relies on its expression.

## Abstract 2

This study reveals a novel role for the calcium-binding protein calreticulin in the survival of cancer cells; downregulation of calreticulin leads to mitochondrial calcium overload and an induction of non-apoptotic cell death. Calreticulin levels inversely correlate with the survival of patients diagnosed with various types of solid cancers.

## Introduction

Calreticulin (CRT) is a multifunctional stress-response chaperone residing mainly in the lumen of the endoplasmic reticulum (ER) in the cell with the ability to control proper protein folding and regulate cellular adhesion [1–6]. The link between CRT and carcinogenesis has mainly been characterized at the level of correlation between augmented CRT expression and disease progression. Studies of multiple solid tumors originating from the skin (melanoma), breast, pancreas, liver, and colon demonstrated that the CRT expression levels were positively correlated with advanced tumor stages and lymph node metastasis [3, 6–8]. At the protein level, expression of CRT was found to be significantly elevated in a number of solid tumors, including glioblastoma, bladder, and ovarian carcinomas when compared with the normal cells of the same tissue origin [7].

A number of other studies have indicated that CRT association with increased metastatic potential is due to its property to positively regulate cell migration and cell survival during anoikis, a form of cell death initiated because of the lack of matrix attachment [9–11]. In fact, CRT has been shown to directly interact with a number of integrin complexes in vitro, including α2β1 to activate cytoskeletal changes required for the acquisition of a migratory phenotype [12, 13]. Under physiologic conditions, critical importance of CRT expression is highlighted by the fact that CRT knockout mice exhibit embryonic lethality with major defects in the heart, brain, and body wall, pointing to an essential role of CRT in tissue growth and development [14–16].

Although “oncogenic” properties of CRT are still under investigation, a number of studies have characterized cell-surface CRT as a factor enabling recognition of cancer cells by professional phagocytes of the innate immune system [7, 17]. CRT displays immunogenic properties when it is translocated to the cell surface in response to a variety of environmental stress signals that include chemotherapeutic drugs, such as anthracyclins and oxaliplatin, as well as Ultraviolet C (UVC) and radiation [7, 17–19]. To resist macrophage-mediated tumor destruction, cancer cells up-regulate Integrin associated protein (CD47), which inhibits their phagocytosis [20–22]. Using patient-derived melanoma cells, we recently demonstrated that blockade of CD47 leads to their efficient phagocytosis and suppression of tumor growth and metastases in vivo [21]. Yet, an important question remained unanswered: what critical role does CRT play during cellular transformation, such that its expression has to be preserved and often times up-regulated by many tumor types, including melanoma, despite its ability to promote immunogenic cell death (ICD). The goal of this study was to identify key properties of CRT that would explain the dependency of neoplastic cells on its expression and characterize molecular events in response to CRT down-regulation. We further explored the clinical importance of these findings by establishing a link between the levels of CRT mRNA and patient survival prognosis using large data sets containing gene expression values individually linked to each cancer-patient survival history.

## Results

### CRT down-regulation causes cell death in multiple solid-tissue–derived malignancies

**Fig 1:**
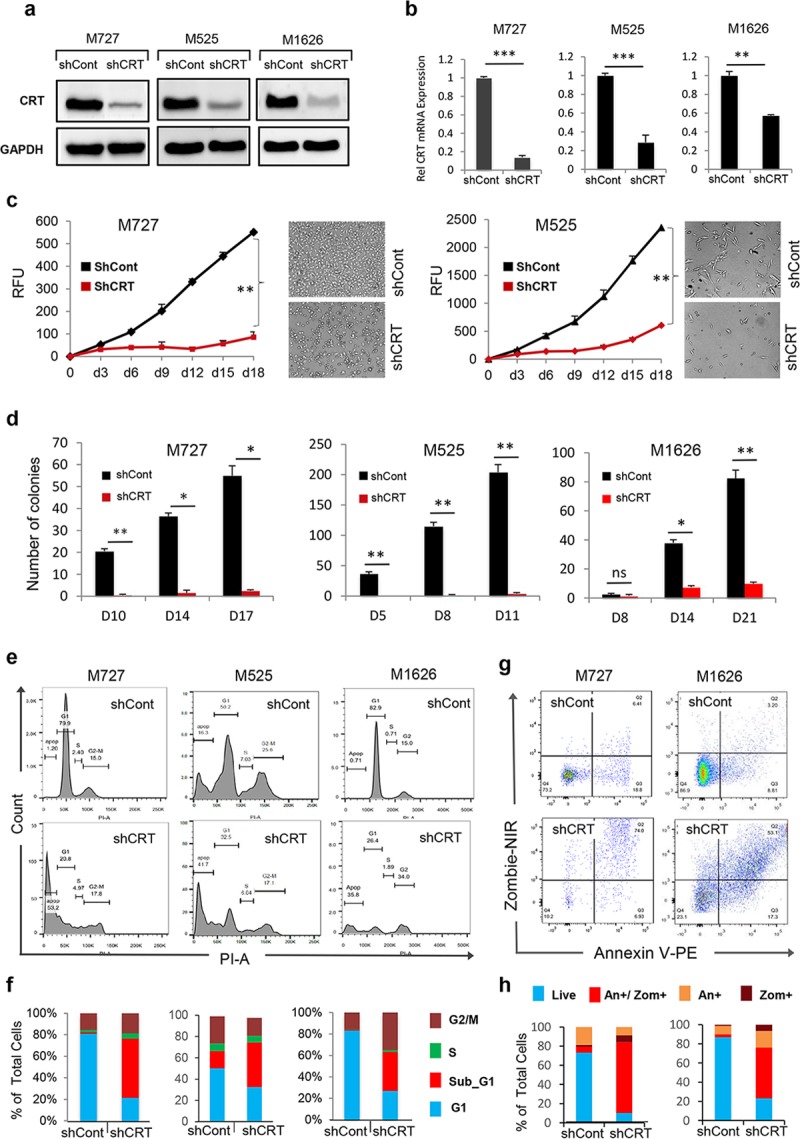
Down-regulation of CRT inhibits malignant properties of melanoma cells by activating cell-death response. Efficient down-regulation of CRT expression at the protein (A) and mRNA (B) levels following transduction of melanoma M727, M1626, and M525 patient-derived cells with the shCRT or shCont lentiviral vectors. Y-axis represents relative fold change in 2^ddcT^ values of CRT mRNA normalized to the control expression of 18 s rRNA. (C) Proliferation kinetics of patient-derived melanoma M727 and M525 cells each transduced with shCRT and shCont as measured by resazurin assay. (D) Number of colonies formed at indicated time periods by M727, M525, and M1626 melanoma cells following CRT knockdown compared with shCont transduced counterparts. (E, F) Cell cycle analysis of indicated melanoma cells using PI stain assay following CRT knockdown. The number of cells in various stages of the cell cycle was quantified measuring the area under the peaks (sub-G1, G1, S, G2−M phases). (G, H) Analysis and quantification of the cell-death phenotype using viability Zombie/Annexin V stain assay following CRT knockdown. Underlying source data can be found in S1 Data. In all panels, unpaired Student *t* test was used to calculate *p*-values (*P < 0.05, **P < 0.005). All error bars indicate mean ± SD. CRT, calreticulin; GAPDH, Glyceraldehyde 3-phosphate dehydrogenase; NIR, Near-Infrared; RFU, Relative fluorescent unit; shCont, short hairpin RNA targeting Control; shCRT, short hairpin RNA targeting Calreticulin.

To examine the role of CRT in melanoma pathogenesis, we employed lentiviral-based short hairpin RNAs (shRNA) expression vectors to down-regulate its expression. Using 2 independent shRNA sequences targeting CRT mRNA, we achieved significant levels of CRT silencing at the mRNA and protein levels in 3 independent patient-derived melanoma cell cultures (Mel727, Mel1626, and Mel525; Figs 1A and 1B and S1A and S1B, lower panel). We then selected shRNA sequence with the most potent silencing efficiency (shCRT/a, hereafter short hairpin RNA targeting Calreticulin [shCRT]), to evaluate the effects of CRT down-regulation on the proliferative capacity of melanoma cells expressing a mutant B-Raf proto-oncogene serine/threonine kinase (BRAF) V600 allele (Mel727) versus cells with wild-type BRAF (Mel525). Growth kinetics of each transduced cell population was measured for a period of 18 days using resazurin assay. The resulting growth curves revealed the significant reduction in the proliferative rate of shCRT-transduced melanoma cells compared with the control (short hairpin RNA targeting Control [shCont])-transduced counterparts independent of the BRAF_V600 status (Fig 1C). We next examined the clonogenic phenotype of melanoma following CRT knockdown by seeding transduced cells at a low density of 10^3^/well in 6-well plates and periodically counted the number of colonies for 21 days. Importantly, we discovered that in addition to their reduced proliferative capacity, shCRT-transduced cells had completely lost their clonogenic potential because they were no longer capable of forming nor supporting colony growth in vitro (Figs 1D and S1C). To determine the underlying cause of tumor cell growth suppression in response to CRT down-regulation, we performed cell-cycle analysis of shCRT and shCont transduced cells. We found that CRT knockdown, using either shCRT/a or shCRT/b targeting sequences, resulted in a significant increase of the subG1 cell population, indicating an active process of cell death (Figs 1E and 1F and S1D). The cell death phenotype of shCRT-transduced cells was further confirmed using viability fluorescent staining and quantitative flow cytometric analysis, revealing a 4- to 7-fold increase of Zombie/AnnexinV -positive cells compared with shCont transduced counterparts in multiple independent patient-derived melanoma cells (Figs 1G and 1H and S1E).

In order to determine that the observed cell death was specific to CRT down-regulation and was not caused by shRNA-mediated off-target effects, we performed CRT rescue experiments. For these assays, we synthesized a full-length CRT cDNA whose amino acid sequence was identical to the cellular variant; however, its DNA sequence contained single nucleotide substitutions, making transcribed mRNA insensitive to shCRT mediated degradation (S2A Fig). This cDNA was cloned into the lentiviral vector (referred as M1CRT) for an efficient transduction of human tumor cells. Next, we co-introduced (via sequential lentiviral infections) M1CRT and shCRT into the target M727 melanoma cells. Two independent control vectors, pLv_zsG and pLv_166, expressing full-length cDNAs for Green fluorescence protein (GFP) were also co-introduced with shCRT into M727 cells. As expected, co-introduction of shCRT with either pLv_166 or pLv_zsG into M727 cells caused dramatic reduction of CRT protein levels (S2B Fig); in sharp contrast, introduction of shCRT along with M1CRT into the same cells no longer resulted in decreased CRT protein levels that remained virtually unchanged compared with the control cells transduced with the combination of shCont and pLv_zsG or pLv_166 (S2B Fig). Importantly, although pLv_166 and pLv_zsG M727 cells ceased to proliferate and underwent phenotypic changes associated with cell death in response to shCRT transduction, M1CRT M727 cells displayed no phenotypic alterations characteristic of dying cells in the presence of shCRT. These results were quantified using the fluorescence-based viability staining assay followed by fluorescence activated cell sorting (FACS) analysis (S2C and S2D Fig) and confirmed that induction of cell death was specific to CRT down-regulation rather than off-target effects caused by shRNA.

**Fig 2:**
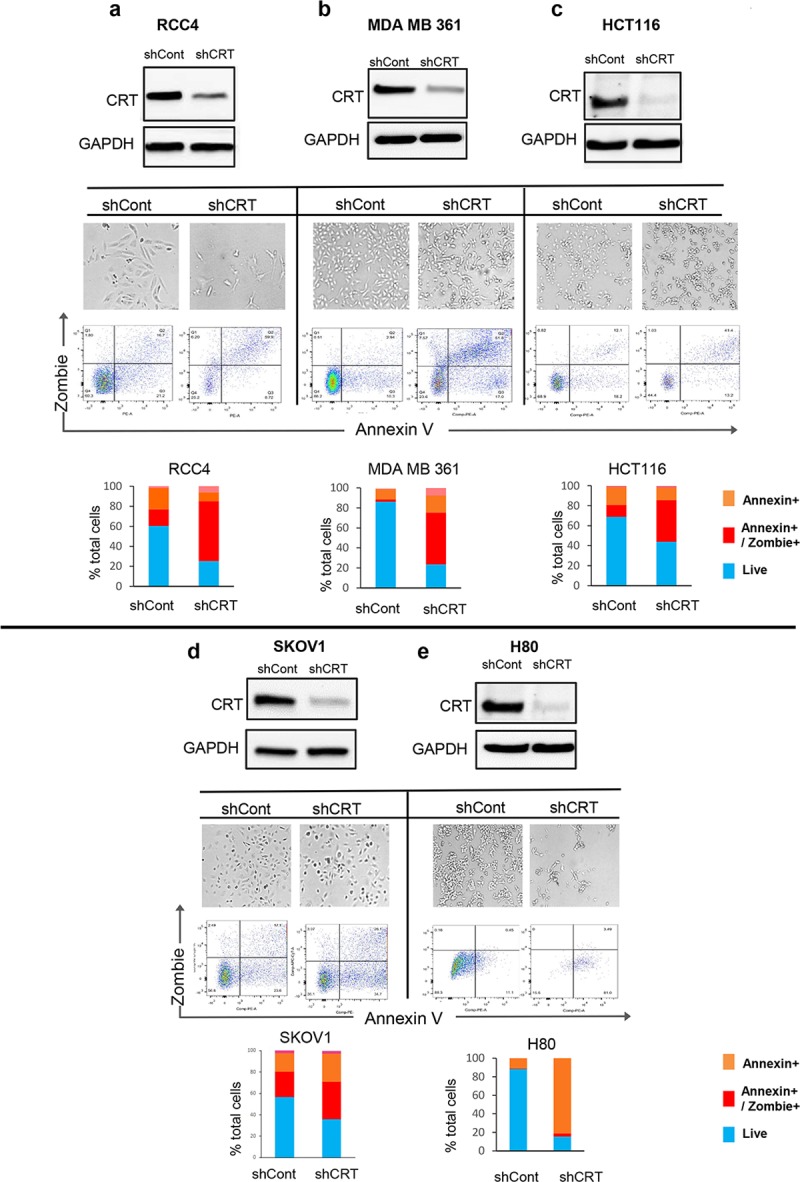
Inhibition of CRT expression initiates the death response in the cells derived from solid-tissue malignancies. Down-regulation of CRT protein (as confirmed by WB) in the cells originating from kidney (A), breast (B), colon (C), ovary (D), and neuronal (E) malignancies causes drastic morphological changes (light microscope at 20× magnification representative images are shown) and a significant increase in the Zombie/Annexin positive cells, all indicative of the active process of cell death. Underlying source data can be found in S1 Data. Bar graphs show percentage of live or dead cells in an indicated tumor types. CRT, calreticulin; GAPDH, Glyceraldehyde 3-phosphate dehydrogenase; HCT116, Human colorectal carcinoma cell line; MB, megabase; MDAMB361, human breast adenocarcinoma cell line; RCC4, Human clear cell renal cell carcinoma cell line; shCont, short hairpin RNA targeting Control; shCRT, short hairpin RNA targeting Calreticulin; SKOV1, Human ovarian carcinoma cell line; WB, Western blot.

To determine if regulation of tumor cell survival by CRT is specific to melanoma or represents a more wide-spread phenomena in cancer, we obtained cells established from multiple solid tumor types, including breast (MDA-MB361), kidney (RCC4), colon (HCT116), ovarian (SKOV1), and neuronal (H80) tumors, and analyzed the effects of CRT knockdown on their viability. Following shCRT transduction, we achieved significant levels of CRT protein silencing in these cells (Fig 2A–2E). Within 7 days post CRT down-regulation, all types of tumor cells lost their ability to proliferate and underwent morphological changes characteristic of cell death, which was further confirmed using fluorescent viability assays and FACS (Fig 2). Subsequent quantitative analysis FACS data demonstrated that CRT down-regulation caused a 2- to 7-fold increase in cell death in all cancers analyzed (Fig 2A–2E). We therefore concluded that control of cell survival by CRT is a general mechanism employed by cancer cells originating from solid tissues.

### Cell death caused by CRT loss is nonapoptotic and executioner caspase independent

**Fig 3:**
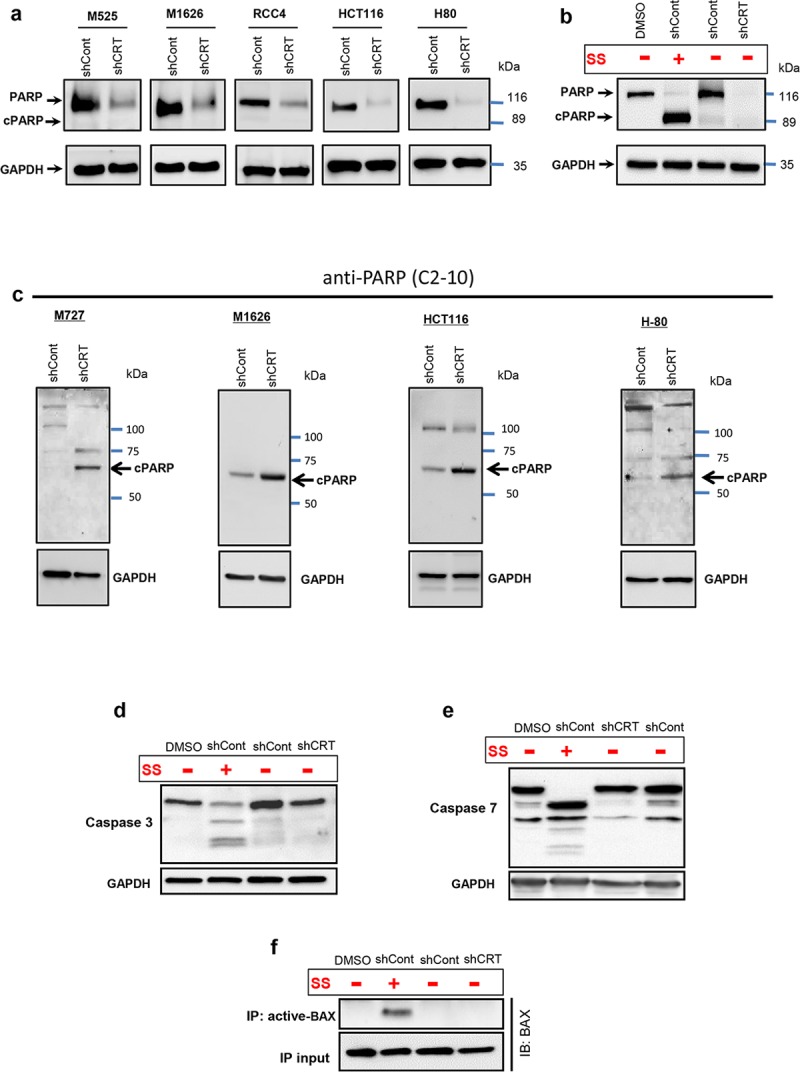
Tumor cell death in response to CRT down-regulation is executioner caspase independent. (A) Western blot analysis of PARP full-length and apoptosis specific cleaved protein fragments (cPARP) in an indicated types of malignant cells following CRT knockdown. (B) Western blot analysis of full-length and cleaved PARP fragment following treatment of cells with apoptosis inducing agent, SS. (C) Western blot analysis of necrosis-specific PARP cleavage fragments using C2-10 anti-PARP mAb in the indicated solid-tissue malignancies transduced with shCRT and shCont. Western blot analysis of the cleaved Caspase-3 (D) and Caspase-7 (E) proteins in the cells following CRT knockdown or SS treatment. (F) Immuno blot of total BAX after IP with antiactive-BAX antibody; 10% of IP input was used as a loading control for BAX. BAX, BCL2-associated X protein; cPARP, cleaved poly ADP ribose polymerase; CRT, calreticulin; GAPDH, Glyceraldehyde 3-phosphate dehydrogenase; HCT, Human colorectal carcinoma cell line; IB, immunoblot; IP, immunoprecipitation; mAb, monocloncal antibody; PARP, poly ADP ribose polymerase; RCC, Human clear cell renal cell carcinoma cell line; shCont, short hairpin RNA targeting Control; shCRT, short hairpin RNA targeting Calreticulin; SS, Staurosporin.

Because cleavage of poly ADP ribose polymerase (PARP) represents one of the major hallmarks of various types of cell death [23], we analyzed the status of this protein in the cells derived from multiple types of solid-tissue malignancies in response to CRT down-regulation. We discovered that in the cells with CRT knockdown, the full-length, 116 kD, PARP protein had been decreased to almost undetectable levels. Intriguingly, ablation of full-length PARP in these cells was not accompanied by the appearance of apoptosis specific, cleaved, 89 kD, PARP fragment (Fig 3A). Using shRNA sequence targeting a different region of CRT mRNA, we observed the same negative effect on the cell viability and levels of full-length PARP protein (S1B Fig upper panel), both of which directly correlated with CRT protein down-regulation levels (S1B Fig lower panel). These results provided evidence that ablation of PARP protein was caused specifically in response to CRT knockdown, rather than an off-target shRNA effect. In order to demonstrate that in our experimental system the levels of full-length, 116 kD, PARP protein can be reduced as a result of its cleavage by activated caspases, giving rise to 89 kD, apoptosis specific, fragment, we treated M727 cells with the classic apoptosis inducing agent, Staurosporin (SS). After isolating protein lysates, we compared PARP status in SS- and DMSO-treated cells, as well as shCRT- or shCont-transduced cells. As a result of SS treatment, we observed efficient cleavage of PARP at the predicted caspase site, resulting in the reduction of full-length PARP (116 kD) protein levels and increased levels of its cleaved product detected as a characteristic 89 kD fragment (Fig 3B). However, the same cells transduced with shCRT lacked both full-length and apoptosis specific PARP fragments (Fig 3B) while undergoing cell death. Complete lack of proapoptotic PARP cleavage pattern led us to hypothesize that CRT knockdown cells were undergoing an alternative form of death, which is accompanied by the different pattern of PARP fragmentation undetectable by apoptosis-specific antibodies. Therefore, we turned our attention to the PARP antibody, C2-10, which was previously raised and characterized to detect alternatively cleaved PARP fragments appearing during nonapoptotic forms of cell death [24]. Using the anti-PARP C2-10 antibody, we analyzed protein lysates from multiple types of malignant cells undergoing cell death in response to CRT down-regulation. Importantly, we discovered that, in fact, PARP was undergoing nonapoptosis-specific cleavage in response to CRT down-regulation, resulting in the accumulation of the approximately 68 kD PARP fragment (Fig 3C). Interestingly, inhibition of Ca^2+^-dependent proteases (calpains) and kinases (CaMKII) that could initiate nonapoptotic PARP cleavage and whose activation was observed during CRT knockdown (S3A and S3B Fig) did not result in any significant rescue the full-length PARP protein (S3C and S3D Fig). Thus, further studies will be required to identify and characterize specific enzymatic proteases involved in the PARP recognition and cleavage during cell-death response due to CRT loss.

To further confirm that CRT down-regulation leads to the nonapoptotic-cell death, we assayed target cells for the activation cleavage of Procaspases 3 and 7, key cysteine proteases involved in the execution steps of apoptosis [25]. As a positive control, we used cells treated with the classic apoptosis inducing agent, SS. Our results indicate that the cell death caused by down-regulation of CRT is in fact independent of executioner caspase activation, because neither Caspase 3 nor 7 undergo cleavage and activation in these dying cells (Fig 3D and 3E). In stark contrast, cells undergoing apoptosis (as a result of SS treatment) displayed significant amounts of cleaved Caspases 3 and 7 as well as activated Bax (Fig 3F).

**Fig 4:**
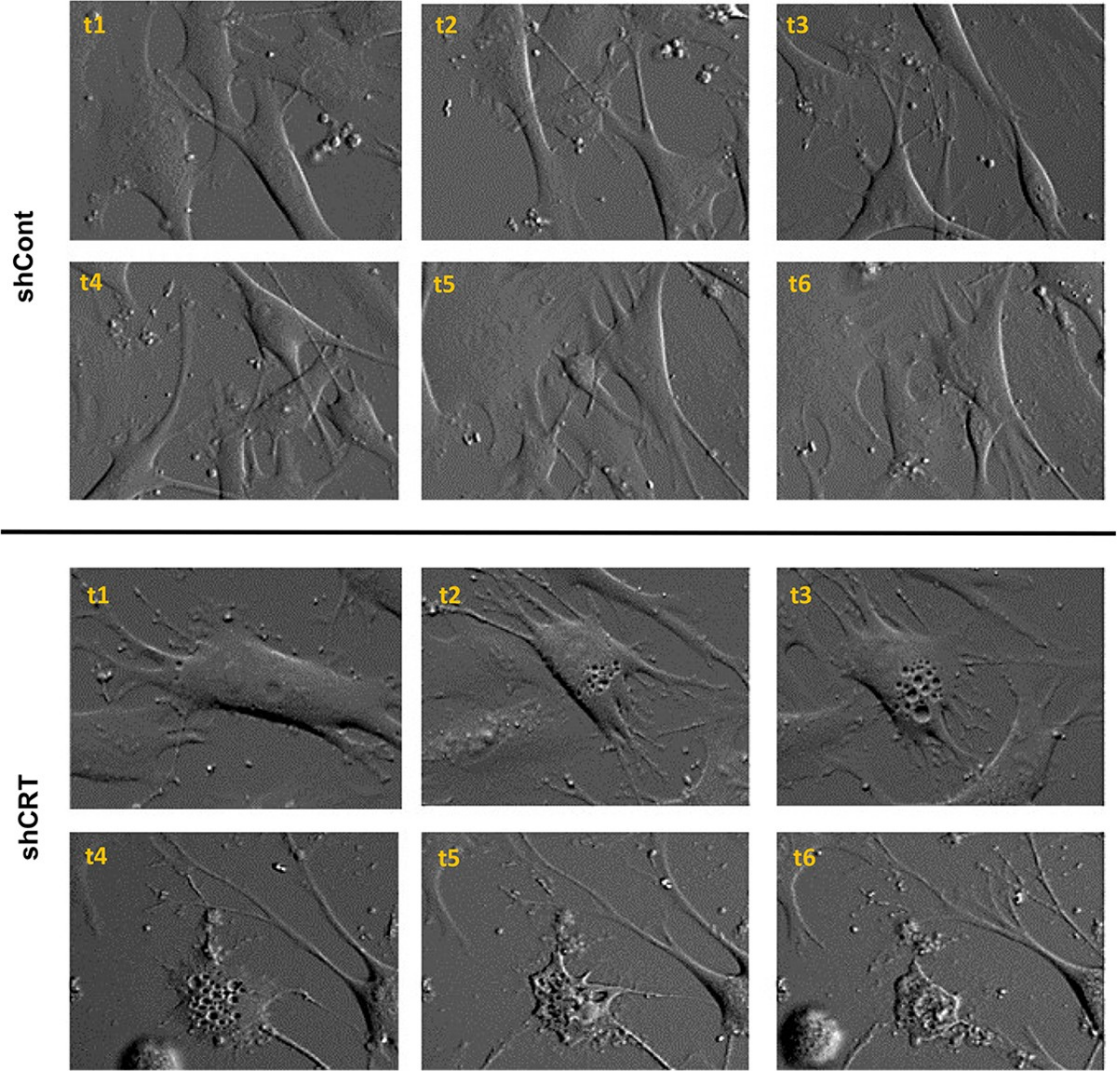
CRT loss initiates nonapoptotic cell-death response leading to the complete cell disintegration. Morphological changes occurring in shCRT and shCont transduced melanoma cells M727 over the period of 72 hours. Bright field image sequence (t1–t6) was taken using 20× objective of the Olympus VivaView FL motorized inverted microscope integrated into TC chamber. CRT, calreticulin; shCont, short hairpin RNA targeting Control; shCRT, short hairpin RNA targeting Calreticulin; TC, tissue culture.

Lastly, we performed a morphologic analysis of the cells dying as a result of CRT knockdown using high-resolution time-lapse microscopy imaging for a period of 72 hours. Our imaging results indicate that CRT down-regulation does not result in the cellular membrane blebbing and formation of characteristic apoptotic bodies but rather is accompanied by extensive vacuolization and appearance of multiple large membrane openings that eventually lead to the cell rupture and complete disintegration (Fig 4).

In summary, the above described results established that the loss of Calreticulin by tumor cells initiates nonapoptotic cell death independent of executioner caspase activity.

### Activated p53 mediates cell-death response during CRT down-regulation

**Fig 5:**
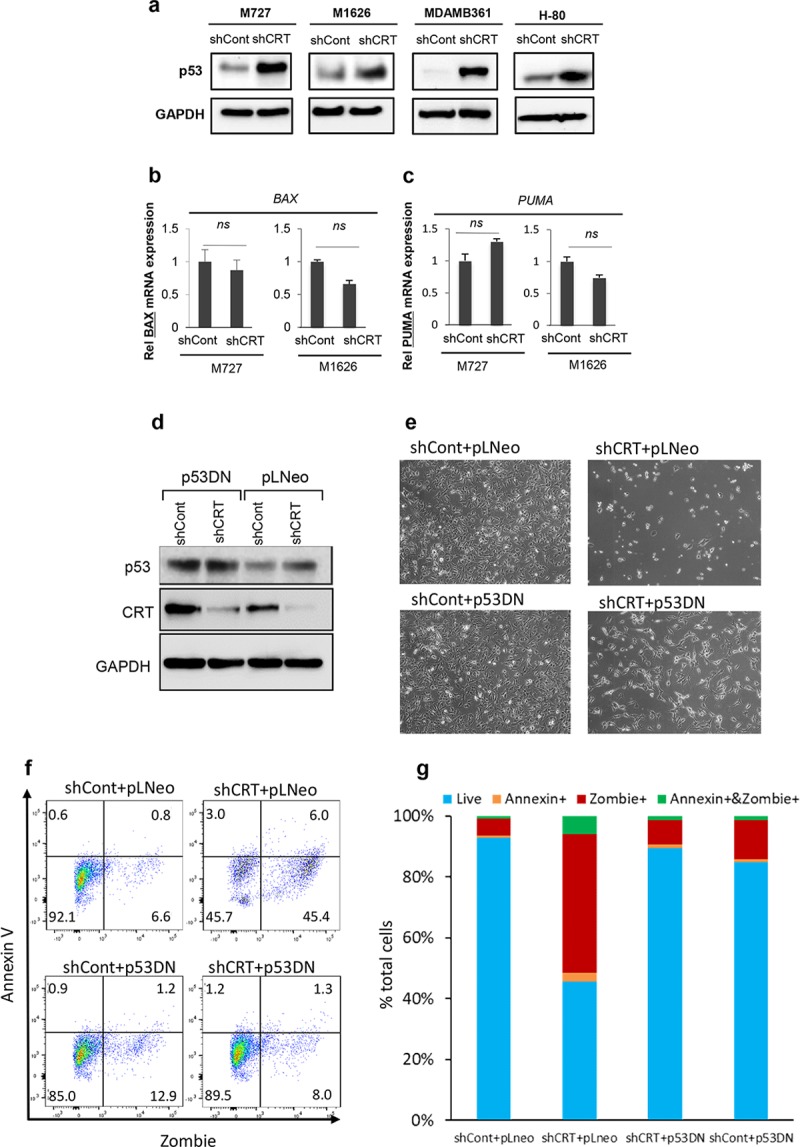
Activated p53 mediates cell death response during CRT down-regulation. (A) Western blot analysis of p53 protein levels in the indicated cell types transduced with shCont or shCRT. Quantification of p53 target genes (B) BAX and (C) PUMA using qRT-PCR and gene-specific primers; Y-axis represent relative fold change in 2^ddcT^ values of an indicated gene mRNA normalized to the control expression of 18 s rRNA. (D) Western blot analysis of p53 and CRT protein levels following transduction of the target cells with p53DN^GSE56^ pLNeo, shCRT, and shCont viral particles. (E) Representative images of the indicated cell populations demonstrating effects of p53 inhibition on the cell survival. (F, G) Flow cytometric analysis and quantification of the cell-death phenotype in the transduced cell populations using Zombie/Annexin V assay. Underlying source data can be found in S1 Data. CRT, calreticulin; GAPDH, Glyceraldehyde 3-phosphate dehydrogenase; MDAMB361, human breast adenocarcinoma cell line; pLNeo, lenti-viral plasmid expressing Neomycin R gene; qRT-PCR, Real-Time Quantitative Reverse Transcription PCR; shCont, short hairpin RNA targeting Control; shCRT, short hairpin RNA targeting Calreticulin.

Regulation of cell-death pathways is often orchestrated by p53, one of the most critical tumor suppressor genes [26–28]. This multifunctional protein is quickly stabilized in response to cellular stress and can induce necrosis-like phenotype under the variety of conditions [29]. Therefore, we hypothesized that CRT down-regulation, which results in the molecular and morphological changes described above, can cause activation of p53, leading to the induction of a nonapoptotic cell death. In order to test this hypothesis, we generated CRT knockdown in multiple human solid-tumor malignancies, as described above, and analyzed them for the expression levels of p53 and its critical downstream targets. Remarkably, we discovered that CRT knockdown in melanoma-, neuroblastoma-, and breast-derived cells caused the significant increase in p53 protein (Fig 5A). We next analyzed canonical p53 downstream target genes known to initiate cell-death response. Interestingly, we did not observe any significant differences between control and CRT knockdown cells in the expression of Bax and Puma, critical proteins that mediate caspase dependent apoptosis induced by p53 (Fig 5B and 5C). These data are consistent with the above observations that CRT down-regulation causes a nonapoptotic type of cell death (Figs 3 and 4).

We next decided to test whether p53 was required for the induction of the cell death in response to CRT down-regulation. To address this question, we utilized a dominant negative p53 inhibitory peptide, p53DN^GSE56^, (spanning amino acids 275–368), which is homologous to the oligomerization domain and effectively suppresses p53 function [30, 31]. Expression of this peptide in target cells renders wild-type p53 protein in an inactive conformation, causing its accumulation in the cytoplasm [30]. After generating stably expressing p53DN^GSE56^ and matching control, lenti-viral plasmid expressing Neomycin R gene (pLNeo), cells, we transduced them with either shCRT or shCont vectors to down-regulate expression of CRT. As expected, introduction of p53DN^GSE56^ caused inactive p53 accumulation in the target cells (Fig 5D). At the same time, shCRT efficiently inhibited CRT expression (Fig 5D) and allowed comparison of cell-death responses between the wild-type p53- and p53DN^GSE56^-expressing populations. Importantly, using fluorescence-based cell-death quantification assay and microscopy analysis 72 hours post shCRT transduction, we determined that p53 blockade significantly enhanced survival of the cells that lost CRT expression (Fig 5E, 5F and 5G). In summary, the above results demonstrate that p53 function is required during nonapoptotic cell death caused by the loss of CRT protein.

### CRT down-regulation leads to the mitochondria Ca^2+^ overload and mitochondria permeability transition pore–dependent cell death

**Fig 6:**
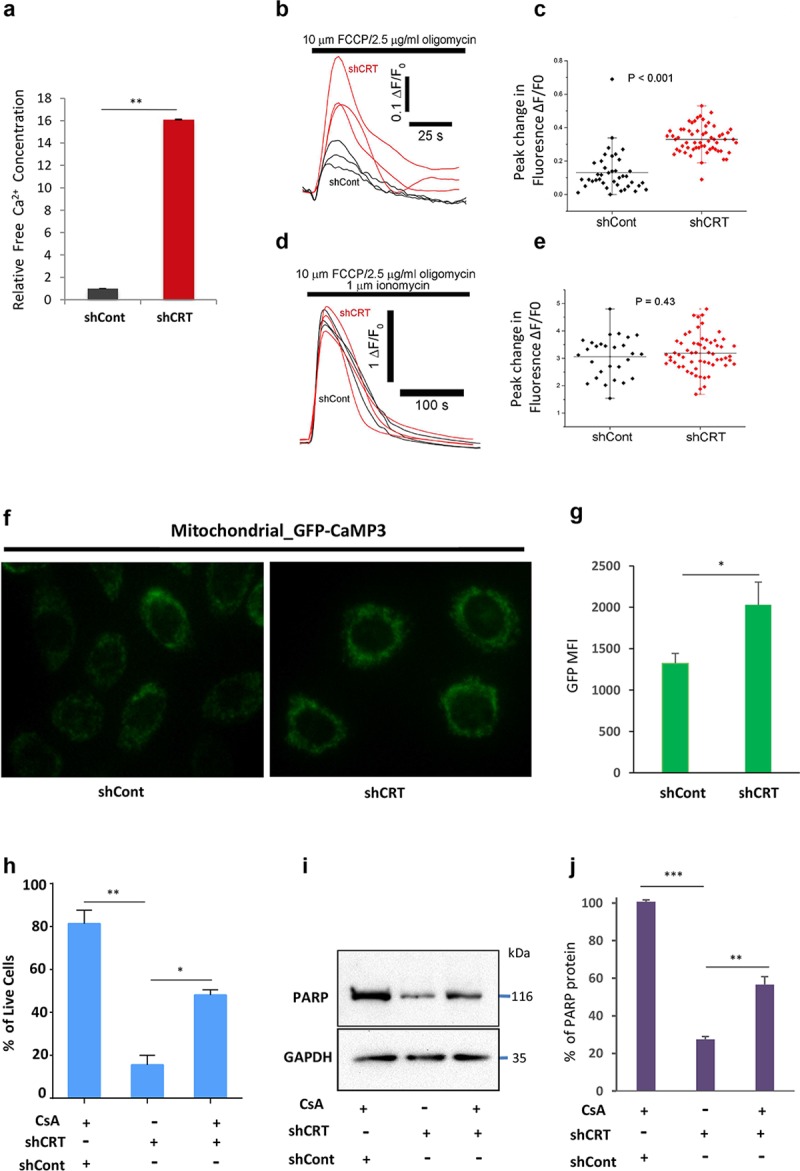
CRT down-regulation leads to the Ca^2+^ overload of mitochondria and induction of CICD. (A) Quantification of free calcium levels in shCont- or shCRT-transduced cells using colometric Ca concentration kit. (B, C) Live whole-cell Ca^2+^ imaging of mitochondrial Ca^2+^ stores, quantified at the single-cell level after administration of FCCP and Oligomycin. (D, E) Live whole-cell Ca^2+^ imaging of mitochondria-independent Ca^2+^ stores, quantified at the single-cell level after administration of FCCP/Oligomycin followed by the addition of Ionomycin. (F) HeLa cell expressing mito- GFP-CaMP3 were transduced with lentiviral particles of shCRT and shCont, and GFP fluoresce was analyzed using Inverted Nikon microscopy system or (G) FACS Instrument, BDCallibur (MFI is plotted on the Y-axis). (H) Viability quantification of the indicated cells after they were grown in the presence of CsA following their transduction with shCRT or shCont. (I, J) Western blot analysis and quantification of PARP full-length protein levels after indicated cells were grown in the presence of CsA. Underlying source data can be found in S1 Data. Unpaired Student *t* test was used to calculate *P*-values (*p < 0.05, **p < 0.005). All error bars indicate mean ± SD. CICD, Caspase-independent cell death; CRT, calreticulin; CsA, cyclosporin A; FACS, fluorescence activated cell sorting; FCCP, Carbonyl cyanide-4-(trifluoromethoxy)phenylhydrazone; GAPDH, Glyceraldehyde 3-phosphate dehydrogenase; GFP, Green fluorescence protein; HeLa, Human cervical adenocarcinoma cell line; MFI, mean fluorescence intensity; mito-GFP-CaMP3, mitochondrial genetically encoded calcium indicator 3, created from a fusion of green fluorescent protein, calmodulin, and M13, a peptide sequence from myosin light chain kinase; PARP, poly ADP ribose polymerase; shCont, short hairpin RNA targeting Control; shCRT, short hairpin RNA targeting Calreticulin.

CRT’s high capacity for Ca^2+^ ions enables it to effectively buffer any changes in an intracellular Ca^2+^ concentration, making it one of the most important storage sinks of Ca^2+^ in the cell [6] [32]. Therefore, we reasoned that knockdown of CRT can lead to the disbalance of the free intracellular Ca^2+^ ion levels and their leakage into the mitochondria, leading to the activation of cell-death machinery. To test this hypothesis, we first compared intracellular free Ca^2+^ ions concentration in shCRT or shCont transduced cells using fluorescent substrate–based calcium quantitation assay. Our data demonstrate that CRT down-regulation causes significant increase in free Ca^2+^ levels inside the target cells (Fig 6A). To determine whether mitochondria organelles, specifically, were affected by these changes, we performed detailed analysis of mitochondria calcium stores. Our measurements were performed at the single-cell level on the population of CRT knockdown cells and matching controls. To measure mitochondria-specific calcium stores, we recorded Ca^2+^ fluorescence signals (in Ca^2+^ free medium) evoked by the administration of the protonophore carbonyl cyanide p-trifluoromethoxy-phenylhydrazone (FCCP) and the ATPsynthase inhibitor, oligomycin (to prevent ATP consumption by the F_1_F_0_-ATPase synthase functioning in reverse mode; Leyssens, 1996). This caused a transient rise in cytosolic Ca^2+^ attributable to release from mitochondrial Ca^2+^ stores that were significantly greater in cells with CRT knockdown compared with parental, scramble-transduced cells (Fig 6B). Quantification of these signals revealed that much higher levels of Ca^2+^ ions are present inside mitochondria following CRT down-regulation (Fig 6C). Next, we repeated the above measurements in the parallel sets of CRT KD and matching control cells to measure Ca^2+^ content in all other organelle stores, including the ER, which is independent of mitochondria. In these assays, cells were first pretreated with FCCP/Oligomycin reagents followed by a challenge with 1 μm Ionomycin to release calcium from the remaining mitochondria-independent stores. Fluorescent Ca^2+^ signals were recorded in Ca^2+^ free medium and indicate that all other organellar stores of CRT KD cells do not display any significant alteration in Ca^2+^ levels compared with matching controls (Fig 6D and 6E). These experiments provided solid evidence that the loss of CRT results in an abnormal increase of Ca^2+^ levels specifically inside mitochondria.

To independently validate our findings that CRT down-regulation leads to the significant increase of mitochondrial Ca^2+^ levels, we used Human cervical adenocarcinoma cell line (HeLa) cancer cell-line expressing genetically encoded calcium indicator 3, created from a fusion of green fluorescent protein, calmodulin, and M13, a peptide sequence from myosin light chain kinase (GFP-CaMP3). Expression of this reporter is specifically targeted to the mitochondria matrix and allows sensitive measurements of Ca^2+^ levels inside mitochondria, based on changes of intensity of GFP fluorescence. Using high-resolution microscopy and flow cytometric analyses, we demonstrate that shCRT transduction of these cells leads to the significant increase of GFP intensity, indicative of augmented mitochondria Ca^2+^ levels compared with the mock, shCont, transduced counterparts (Fig 6F and 6G).

Following these observations, we reasoned that the cytotoxic effects of CRT down-regulation can be caused by the mitochondria Ca^2+^ overload leading to the formation of mitochondria permeability transition pore (mPTP) and subsequent induction of nonapoptotic cell death. To test this hypothesis, we set out to determine whether mPTP inhibitor, Cyclosporin A (CsA), can rescue cell survival in response to CRT down-regulation. Briefly, M727 melanoma cells were transduced with shCRT and shCont lentiviral particles, as described above, to generate CRT knockdown and matching controls. These cells were then seeded on 6-well plates at the density of 2 × 10^4^ per well, and the CsA or DMSO reagents were administrated into the cell culture media at a concentration of 2.5 μM for 24 hours. At the end of the incubation time period, cell viability was analyzed using Zombie/Annexin staining assay, which was then quantified using a BD Biosciences fluorescence activated cell sorting (BDFACS) instrument. Our results demonstrate that inhibition of mPTP leads to the partial, yet significant, increase in cell survival following CRT down-regulation (Fig 6H). Importantly, CsA treatment also rescued PARP full-length protein levels in the CRT KD cells to the extent that directly correlated with increased cell survival compared with the control counterparts (Fig 6I and 6J). In summary, the above results identify the critical role of CRT in preventing mitochondrial Ca^2+^ overload and MPTP induced nonapoptotic cell death.

### CRT expression inversely correlates with survival of patients diagnosed with multiple types of solid cancers

**Fig 7:**
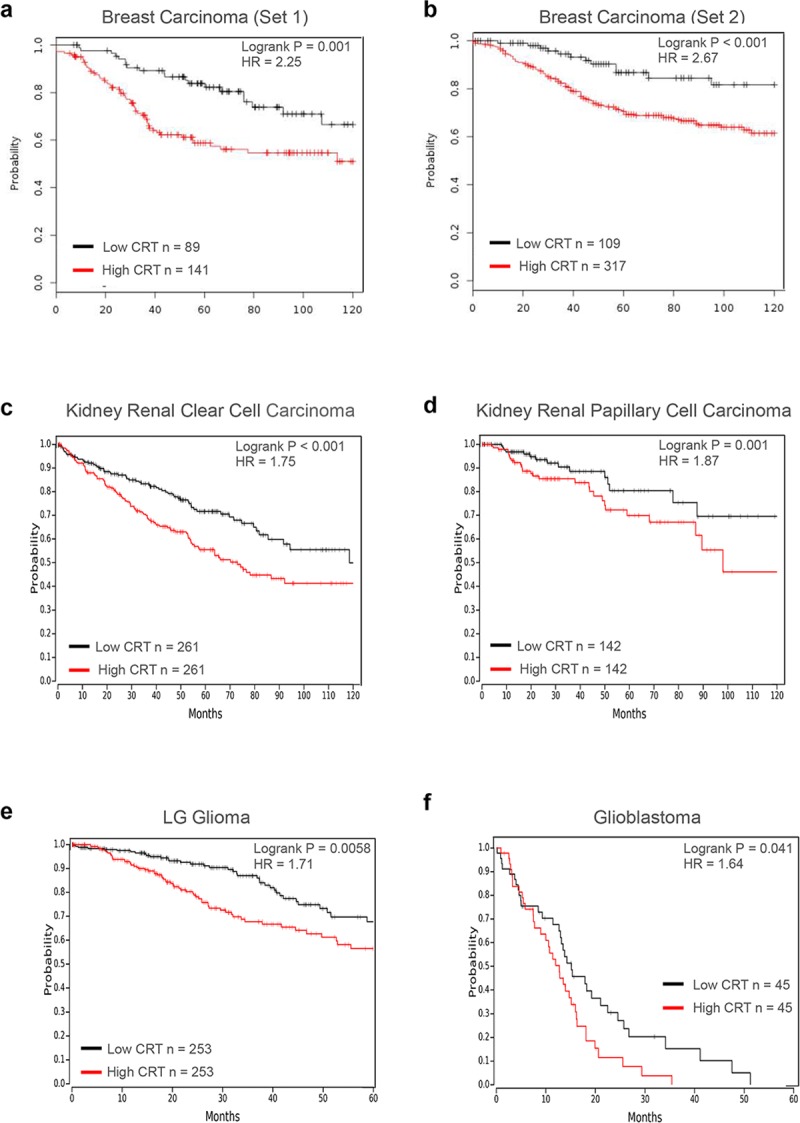
CRT expression inversely correlates with patients survival diagnosed with multiple types of solid cancers. Kaplan-Meier survival analysis is shown for patients diagnosed with breast- (A–B), kidney- (C–D), and brain- (E–F) derived malignancies. Patients were stratified into high and low groups based on median CRT expression (C, D, E) or using an optimal cut-off approach (A, B, F), and overall survival is plotted. Clinical data sets were downloaded from either Gene expression Omnibus (A: GSE 21653, B: EM5BC) or TCGA (–-F) using online database tools [33, 34]. *p*-Values were determined using log-rank test; HR was determined using a univariant Cox regression model; values are shown for the relationship of outcomes to dichotomous expression of CRT. CRT, calreticulin; HR, hazard ratio; TCGA, The cancer genome atlas.

Given the critical importance of CRT expression in the survival of malignant cells, we evaluated the clinical significance of these findings. Using gene expression data sets individually linked to each patient outcome, we evaluated CRT mRNA expression levels and how they correlate with each patient survival. Large cohorts of patients diagnosed with diverse solid-tumor malignancies, including breast, kidney, and brain, were stratified into low and high subgroups based on CRT expression, and their survival was evaluated over a period of 5 or 10 years using Kaplan-Meier analysis [33, 34]. Each data set contained patients diagnosed at different stages of the disease progression and undergoing various types of therapeutic treatments. Despite these differences, high CRT expression was consistently associated with the worst survival outcome in all patient groups analyzed: breast carcinoma S1 (*N* = 230; log *p* = 0.0001) and S2 (*N* = 426; log *p <* 0.0001; Fig 7A and 7B), kidney renal clear cell carcinoma (*N* = 522, log *p <* 0.0001), kidney renal papillary cell carcinoma (*N* = 284, log *p =* 0.0483; Fig 7C and 7D), low-grade glioma (*N* = 306 log *p <* 0.0001), and glioblastoma (*N* = 90, log *p =* 0.0417; Fig 7E and 7F). These data indicate that high level of CRT expression is clinically relevant to the progression and aggressiveness of the above mentioned types of tumors and represents an important therapeutic target. Yet, it needs to be noted that patient-specific analysis of CRT expression described above is limited to the mRNA levels and cannot directly predict redistribution of CRT protein within the cell, including its translocation to the surface membrane, which may influence patient therapeutic response.

## Discussion

CRT represents one of the most critical Ca^2+^ storage proteins because over 50% of the entire Ca^2+^ in the cell is bound to this chaperone [2]. CRT knockout mice have been generated in the past and display embryonic lethality due to the defects in the intracellular Ca^2+^ homeostasis that affects heart development [14, 15, 35]. In our current study, we reveal that CRT plays a major role in the cancer cell survival, and its down-regulation induces a nonapoptotic type of cell death, independent of executioner caspase activity. Significantly, we demonstrated that CRT knockdown causes disbalance of intracellular Ca^2+^, resulting in the considerable mitochondrial Ca^2+^ overload. The integrity of the mitochondrial membrane is a key factor in determining the balance between a life/death outcome, and calcium plays an important role in this decision [32, 36–38]. Formation of the mitochondria outer membrane pores (MOMPs) under transient Ca^2+^ fluctuations can cause the release of *Cytochrome c*, activation of the caspase machinery, and induction of apoptosis. However, a sustained increase in Ca^2+^ levels can lead to the formation of the mPTPs and induction of nonapoptotic cell death [39, 40]. In our experiments, we demonstrated that inhibition of mPTP formation using cyclophilin D inhibitor, CsA, can significantly enhance cell survival under conditions of CRT loss, revealing critical involvement of mPTP in the initiation of the cell-death response.

**Fig 8:**
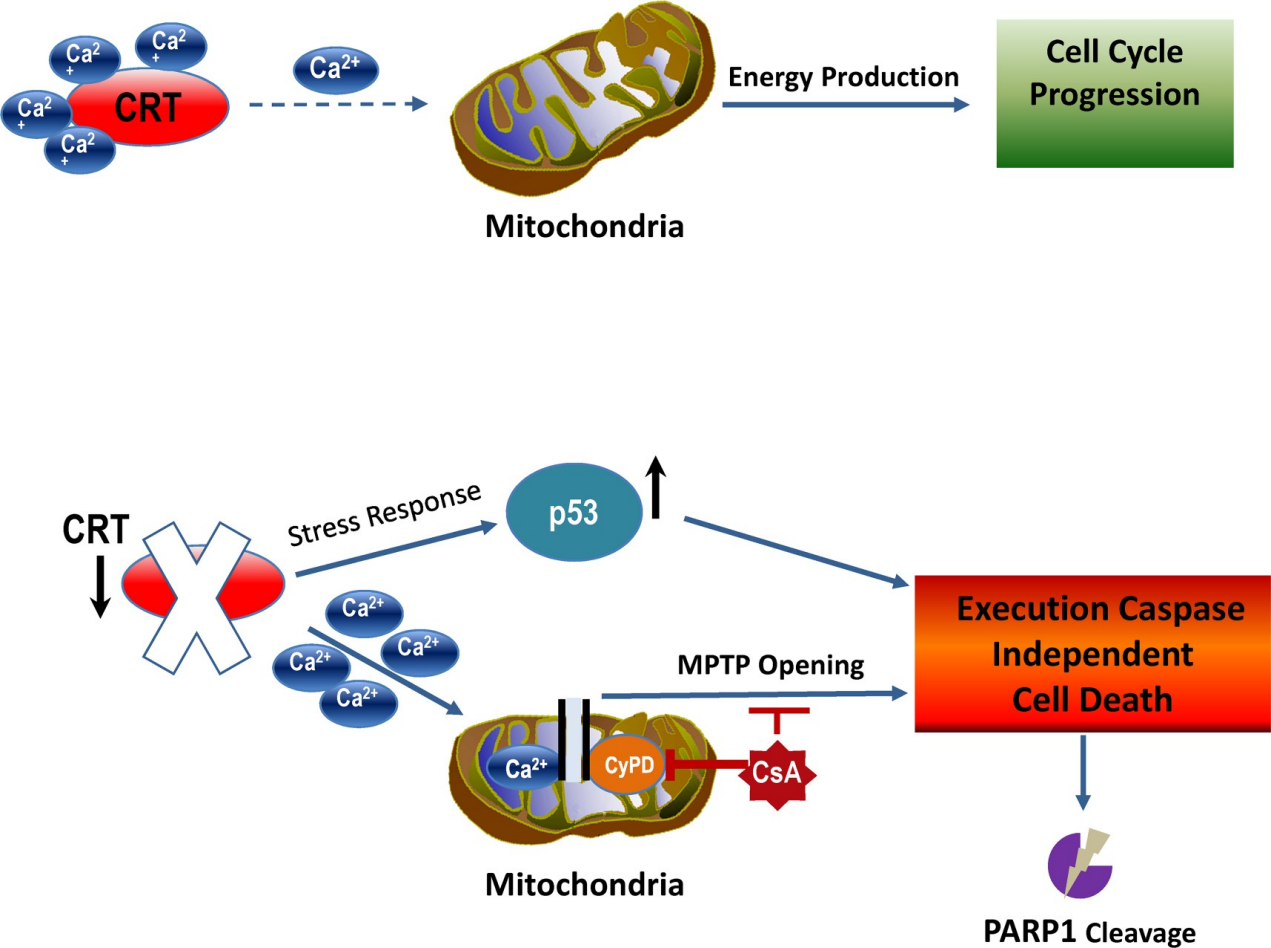
General model of the CRT prosurvival function in the cell. CRT, calreticulin; mPTP, mitochondria permeability transition pore; PARP, poly ADP ribose polymerase.

Importantly, in our study, we discovered that CRT down-regulation causes activation of the major stress response protein, p53. Yet, unlike in many other cases, induction of the p53 dependent gene targets involved in the MOMP formation, such as BAX and PUMA, was not observed in the cells that lost CRT expression. This explains the lack of key executioner apoptotic caspase (Cas-3 and Cas-7) activation and nonapoptotic PARP cleavage pattern (Fig 3). Previous studies implicated p53 and augmented Ca^2+^ levels in the regulation of nonapoptotic cell death either through transactivation of alternative gene targets or by directly participating in the mPTP formation [29, 41–43]. Thus, it was demonstrated that p53 transcriptional activity is required for the induction of necrosis-like cell death by up-regulating expression of various gene targets, which, depending on the stress-inducing factors and the cell type, include Apoptosis Inducing Factor (AIF), Cathepsin Q, necrosis related factor (NRF), AMP-activated protein kinase (AMPK), Tuberous Sclerosis Complex 2 (TSC2), and others [44–47]. On the other hand, evidence exists that active p53 can directly translocate to the mitochondria matrix during ischemia and oxidative stress and bind to cyclophilin D, facilitating mPTP formation and induction of caspase-independent cell death [40, 41]. In all of the above scenarios, p53 activity along with sustained Ca^2+^ fluctuations do not lead to the *Cytochrome c* release and executioner caspase activation but rather result in the necrotic phenotype followed by the complete cell disintegration [41, 48, 49]. Our molecular and morphological analysis of cell death in response to CRT loss supports this model. Importantly, we uncovered the critical role of p53 in this process by using dominant negative peptide known to fully suppress its function, including an ability of p53 to act as a transcription factor [30, 31]. In our experiments, inhibition of p53 lead to the significant increase in cell survival following CRT down-regulation (Fig 5). This signifies that p53 is not a mere marker up-regulated as a result of the cellular stress in the absence of CRT but directly participates in the induction of nonapoptotic cell death under these conditions. Therefore, we propose a mechanism in which CRT down-regulation results in both activation of p53 and disbalance of mitochondrial Ca^2+^, followed by mPTP opening. Cooperation of these molecular events accompanied by the full-length PARP cleavage leads to the necrotic type of cell death (Fig 8). Identification and use of the p53 inhibitors that discriminate between its ability to induce target gene expression or block protein-protein interactions directly involved in mPTP formation will further delineate p53 function in this process. It will also allow molecular characterization of the downstream proteins, regulated by p53 and Ca^2+^, which execute the death pathway in response to CRT down-regulation.

Significantly, the data presented in this report helps explain the paradox previously established in the field by the studies that link CRT expression to tumor progression and cellular transformation, whereas others had shown that CRT can initiate an antitumor immune response diminishing tumor growth [3, 7, 17, 50]. These 2 phenomena are not mutually exclusive with the discovery of the intracellular prosurvival function of CRT required during neoplastic cell growth described here. Based on this model, a cell undergoing malignant transformation would evolve according to either one of the following scenarios: one is to preserve or augment CRT expression, enabling greater adaptation to the changes in cellular metabolism and calcium fluctuations, as well as, enhancing cell migratory properties leading to increased metastasis. At the same time, these cells would adapt the mechanism against CRT-mediated immune recognition and distraction that includes overexpression of immune-inhibitory antigens or prevent CRT from being translocated to the cell surface [17, 51, 52]. In fact, identification of CD47 as a potent antimacrophage signal co-expressed in the cells with high levels of CRT in multiple types of hematologic and solid malignancies provides biological evidence of this scenario [20–22, 53]. In the second model of tumor progression, up-regulation of proteins with high Ca^2+^ binding capacity, such as Calnexin or Binding immunoglobulin protein (BiP), as well as mutations in p53 sensory cell death machinery can account for the tumor types that had to down-regulate CRT expression early in their evolution because of their inability to resist its proimmunogenic function. In this regard, it is interesting to note that in the patients diagnosed with non–small-cell lung cancer in which, in general, p53 mutation rate is close to 70%, low cell surface CRT levels were shown to be associated with higher tumor aggressiveness [54].

In our study, the clinical significance of the prosurvival function of CRT was established by revealing a correlation between high levels of CRT mRNA expression and poor survival prognosis for the patients diagnosed with breast, kidney, and brain malignancies. It is important to note that high levels of CRT expression in more aggressive tumors may also be due to the augmented requirement of CRT chaperone function. CRT structure has been functionally mapped to contain N-terminal region capable of interacting with multiple proteins, including integrins, surface receptors, transporters, and others, to ensure their proper assembly and function [1, 55, 56]. In fact, increased levels of CRT in the ER may allow cancer cells, which often display elevated rates of protein synthesis, to facilitate their accurate folding and avoid activation of the unfolded ER stress response pathways detrimental for cell growth. Also, previous studies have demonstrated that elevated levels of CRT can positively regulate an activity of the Phosphoinositide 3-kinase (PI3K)-Protein kinase B (AKT) singling axis underlying tumor cell resistance to anoikis and increased migration, resulting in an enhanced metastatic potential [5, 57]. In fact, when we analyzed shCRT and matching control cells, we observed a significant reduction in AKT phosphorylation as a result of CRT down-regulation (S4 Fig). Additional studies will be required to elucidate the precise role of CRT during metastatic progression.

Collectively, our results provide a biological explanation to the sustained presence and up-regulation of CRT during cellular transformation despite its immunogenic properties. Further research will need to be undertaken to develop inhibitors specifically targeting intracellular CRT in order to diminish tumor cell viability and significantly increase the likelihood of a positive therapeutic outcome.

## Materials and methods

### Cell cultures, growth, and treatment conditions

Human melanoma patient-derived cells M525, M727, and M1626 were obtained as previously described by Boiko and colleagues [58, 59]. RCC4, SKOV1, MDA-MB 361, and HCT116 were kind gift of Dr. Razorenova lab; H80 (U-251) cells were a kind gift of Dr. Bota lab; Hela_GFP-CAMP3 cells were generated in Dr. Green Lab. M727, RCC4, MDA-MB 361, HeLa, and MCF7 cells were grown in Dulbecco’s Modified Eagle’s Medium (DMEM; Corning, Corning, NY) supplemented with 10% fetal bovine serum (FBS; Omega Scientific, Tarzana, CA) and Penicillin/Streptomycin at 37°C, 5% CO_2_. M525 cells were cultured in RPMI 1640 medium (Corning, Corning, NY) supplemented with 5% FBS. M1626, HCT116, SKOV1, and H80 cells were maintained in RPMI 1640 medium supplemented with 10% FBS. For the SS experiments 2 × 10^5^ cells were seeded in p60 plates and incubated in the growth media containing 2 μM of SS (Enzo Lifesciences, Farmingdale, NY) for 3 hours. For CsA treatment, experiments cells were then seeded on 6-well plates at the density of 2 × 10^4^ per well, and the CsA reagent was administrated into the cell culture media at a concentration of 2.5 μM for 24 hours.

### shRNA-mediated silencing of CRT

Permanent CRT knockdown is achieved using the shRNA sequences (5ʹ-CGTCTACTTCAAGGAGCAGTT-3ʹ, 5ʹ-GCACGGAGACTCAGAATACAA-3ʹ) homologous to CRT mRNA that are cloned into the lentiviral vector pLKO.1_Puro (Sigma, St. Louis, MO), allowing for stable gene silencing in the target cells after their lentiviral transduction. Self-inactivating replication incompetent viral particles containing shCRT sequence are produced in virus packaging cells (HEK293T) by co-transfection of pLKO.1_Puro_shCRT plasmid with compatible packaging plasmids, pCMVΔR8.2 and pVSV-G, using Viafect reagent (Promega, Madison, WI). Culture supernatants containing infectious viral particles were then collected for the period of 72 hours in 8 to 10 hour intervals. Target cells are seeded on p60 cell culture dishes at the density of 10^5^ and infected with shCRT lentiviral particles 3 to 6 times over the period of 4 days, after which cells are reseeded to p100 dishes at the density 5 × 10^4^ to 10^5^, and the phenotypic changes associated with CRT down-regulation start to appear on approximately day 6.

### Cell growth and colony formation assays

Infected cells (10^4^) were seeded in a 6-well plate in duplicates and grown at 37°C, 5% CO_2_. Conditioned media were collected at each indicated time point and incubated with resazurin sodium salt (Promega, Madison, WI) in a black 96-well tissue culture plate at 37°C, 5% CO_2_ for 30 minutes. Fluorescence and absorbance were subsequently measured using a plate reader (Biotech Synergy HT). For adherent colony formation assays, 10^3^ cells were seeded in 6-well plates and cultured at 37°C, 5% CO_2_. Numbers of colonies were counted every 3 days for 2 weeks under light microscope. After 2 weeks, the plates were washed with PBS, fixed with MetOH, and stained with crystal violet.

### Cell viability and cell cycle analysis

Cells were trypsinized and centrifuged at 300*g* for 5 minutes at 4°C and washed with 0.5 mL of cold PBS. The single-cell suspensions were washed in HBSS and counted. Cells (10^5^) were resuspended in 100 μL of HBSS/2% FBS and stained with either with Annexin V-PE (BD Biosciences, Franklin Lakes, NJ) or Zombie-NR-APC_Cy7 (BioLegend, San Diego, CA). Stained cell suspensions were analyzed using BD LSR flow cytometer. Underlying raw FACS data can be found online at the http://flowrepository.org/experiments following the links for each respective figure of the manuscript. For cell cycle analysis, 1 × 10^5^ to 2 × 10^5^ cells were washed in PBS, fixed with 70% ethanol/PBS overnight and stained with Propidium Iodide (Life Technologies, Carlsbad, CA) for 30 minutes at room temperature before flow cytometric analysis as described above. All flow cytometry data were analyzed using FlowJo software (Tree Star).

### Ca^2+^ quantification and whole-cell live Ca^2+^ imaging

Intracellular calcium was quantified using colorimetric calcium detection kit (Sigma-Aldrich); briefly, cells were trypsinized and gently homogenized in an assay buffer by passing through a syringe. After centrifugation, the supernatant was collected in a 96-well plate and analyzed for calcium concentration by measuring absorbance using a plate reader (Biotech Synergy HT). Final calcium concentrations were calculated according to the manufacturer’s protocol. For live cell Ca^2+^ imaging, cells seeded in glass bottom dishes were incubated in culture medium containing Cal-520 AM for 45 minutes at 37°C in a humidified environment with 95% air and 5% CO_2_. Following this, the bath solution was changed to a HEPES-buffered saline (2.5 mM CaCl2, 120 mM NaCl, 4 mM KCl, 2 mM MgCl2, 10 mM glucose, 10 mM HEPES), and cells were subsequently kept at room temperature. For Ca^2+^ free perfusate, Ca^2+^ was exchanged with 1 mM EGTA. For FURA-2 imaging, changes in [Ca^2+^]i was determined using the InCyt Im2 Ratio Imaging System (Intracellular Imaging Inc., Cincinnati, OH) with a ×40 (numerical aperture = 1.3) oil objective. Fura-2 was excited alternatively at 340 and 380 nm with emitted fluorescence (λ = 510/40 nm) imaged using a COHO cooled CCD camera. Changes in [Ca2+]i are presented as ratio signals. For Cal-520 imaging, [Ca^2+^]i changes were imaged using a Nikon Eclipse microscope system (Nikon, Melville, NY) with a ×20 (numerical aperture = 1.30) air objective. Cal-520 fluorescence was excited by 488-nm laser light, and emitted fluorescence (λ > 510 nm) was imaged using an sCMOS ORCA-flash4.0LT Camera (Hamamatsu, Japan). Fluorescence signals are expressed as a ratio (ΔF/F0) of changes in fluorescence (ΔF) relative to the mean resting fluorescence at the same region before stimulation (F0). FCCP and oligomycin reagents were purchased from Cayman Chemical (Chicago, IL).

### Western blot

Cells were lysed with RIPA buffer, and protein concentration was determined by BCA assay (Pierce, Waltham, MA). Protein lysates were separated by SDS–PAGE, transferred to nitrocellulose membranes (Bio-Rad), blocked with 5% milk/TBS, and probed with antibodies in 4°C overnight. The following antibodies were used for Western blot: mouse anti-CRT (Enzo Life Sciences, Farmingdale, NY), mouse anti-GAPDH (Fitzgerald, Acton, MA), rabbit anti-caspase 3 (Cell Signaling Technology, Danvers, MA), rabbit anti-caspase 7 (Cell Signaling Technology, Danvers, MA), rabbit anti-BAX antibody (abcam, Cambridge, MA), rabbit anti-PARP (Cell Signaling Technology, Danvers, MA), mouse anti-PARP C2-10 (Trevigen, Gaithersburg, MD), mouse anti-p53 (Santa Cruz, Dallas, TX), rabbit anti-CaMKII and anti-phospho CaMKII (Cell Signaling Technology, Danvers, MA).

### Calpain protease assay

Calpain activity was measured using Calpain-Glo protease assay kit (Promega, Madison, WI) according to the manufacturer's instructions. Cells were counted, lysed on ice, centrifuged, and incubated with Suc-LLVY-Glo substrates and reagent for 30 minutes at room temperature in the dark. After incubation, luminescence emission was measured at indicated time points using Sirius Luminometer version 3.2.

### Quantitative real-time PCR

Total RNA was extracted from cancer cells using Trizol reagent (Life Technologies, Carlsbad, CA). The mRNAs were reverse transcribed into cDNAs using Verso cDNA synthesis kits (Life Technologies, Carlsbad, CA), followed by real-time PCR using LightCycler FastStart DNA Master SYBR Green I and LightCycler 780II (Roche Diagnostics, Basel, Switzerland). Gene-specific primer sets are summarized in S1 Table. Values were normalized against GAPDH or 18 s using the 2^-ΔΔC^_T_ method (Kenneth J. Livak and Thomas D. Schimittgen). Data are shown as means and standard deviations (SD) with unpaired Student *t* test with *p* < 0.05.

#### Time lapse microscopy

shCRT or shCont transduced cells were plated on the glass bottom dish (In Vitro Scientific, Mountain View, CA) coated with fibronectin (100 μg/mL in PBS). Cells were maintained in a complete media at 37°C and 5% CO_2_ in TC chamber. Live imaging was performed using Olympus VivaView FL motorized inverted microscope integrated into TC chamber. Images were taken using 20× objective every 6 minutes for a period of 72 hours. Image data were transferred, processed, and analyzed using Imaris software.

#### Analysis of cancer patient survival based on CRT expression levels

Clinical and gene expression data linked to individual patient survival follow up were downloaded and analyzed using KM Plotter[33] (for breast cancer patients GSE 21653 and EM5BC sets) and OncoLnc [34] (for kidney and brain cancer patients TCGA sets) software tools. Briefly, patients were stratified into high and low groups based on median CRT expression (KIRC, KIRP, and LGG) or using optimal cut-off approach (Breast and GBM cancers), and Kaplan-Meier analysis was performed for overall survival. *p*-Values were determined using log-rank test; confidence interval (CI) was set at 95%, hazard ratio (HR) was determined using a univariant Cox regression model; values are shown for the relationship of outcomes to dichotomous expression of CRT.

## Supporting information

S1 Fig shRNAs targeting 2 different mRNA regions of CRT down-regulate its protein expression and induce necrotic cell death. (A) Quantification of CRT mRNA levels in shCRT/a, shCRT/b, and shCont transduced cells using qRT-PCR and CRT-specific primers. (B) Analysis of CRT and PARP 1 protein levels in shCRT/a, shCRT/b, and shCont transduced cells using Western blot. (C) Representative images of the colony numbers and sizes formed by the shCont or shCRT-transduced melanoma cells (M727, M525, and M1626) following their fixation and crystal violet staining. (D) Cell cycle analysis of shCRT/a, shCRT/b, and shCont transduced cells after they were stained with PI and analyzed using flow cytometry. The number of cells in various stages of the cell cycle was quantified by measuring the area under the peaks (sub-G1, G1, S, G2−M phases). (E) Analysis and quantification of cell death phenotype in shCRT/a, shCRT/b, and shCont transduced cells using Zombie/Annexin V stain and flow cytometry. Underlying source data can be found in S1 Data. CRT, calreticulin; PARP, poly ADP ribose polymerase; PI, propidium iodide; qRT-PCR, Real-Time Quantitative Reverse Transcription PCR; shCont, short hairpin RNA targeting Control; shCRT, short hairpin RNA targeting Calreticulin; shRNA, short hairpin RNA.
(TIF)

S2 Fig shCRT phenotype rescue with the full-length M1_CRT cDNA. (A) Sequence alignment of shCRT, wild-type CRT, and shCRT insensitive M1, CRT mutant. (B) Analysis of CRT protein levels in the target cells transduced with the combination of indicated plasmids and assayed using WB and CRT-specific antibodies. (C) Quantification of cell viability using Annexin/Zombie fluorescent assay following their transduction with the combination of indicated plasmids. (D) Representative FACS plots of the viability stains. Underlying source data can be found in S1 Data. CRT, calreticulin; FACS, fluorescence activated cell sorting; shCRT, short hairpin RNA targeting Calreticulin; WB, Western blot.
(TIF)

S3 Fig Activation of Ca^2+^ dependent enzymes in shCRT-transduced cells. (A) Protein level analysis of phospho- and pan- CaMKII using Western blot in the indicated solid tumor cells following their transduction with shCont or shCRT. (B) Quantification of the Calpain activity in shCont- or shCRT-transduced cells at indicated time points using Calpain-Glo assay. Unpaired Student *t* test was used to calculate *P*-values (**P < 0.005, ***P < 0.0005). All error bars indicate mean ± SD. Analysis of full-length PARP protein levels using Western blot following incubation of the indicated cells with CI (C) or CamKII inhibitor, KN95 (D). Underlying source data can be found in S1 Data excel table. CI, Calpain inhibitor; PARP, poly ADP ribose polymerase; shCont, short hairpin RNA targeting Control; shCRT, short hairpin RNA targeting Calreticulin.
(TIF)

S4 Fig Reduced AKT phosphorylation due to CRT down-regulation. Analysis of AKT-P^Ser473^, total AKT, and CRT protein levels using WB and respective antibodies following transduction of target cells with shCRT, CRT-nontargeting shRNAs (shU1 and shU2), and shCont. AKT, Protein kinase B; CRT, calreticulin; shCont, short hairpin RNA targeting Control; shCRT, short hairpin RNA targeting Calreticulin; shRNA, short hairpin RNA; WB, Western blot.
(TIF)

S1 Data Raw data underlying figures provided in the manuscript. (XLSX)

S1 Table (PDF)

## Abbreviations

AIF: Apoptosis Inducing Factor
AKT: Protein kinase B
AMPK: AMP-activated protein kinase
BAX: BCL2-associated X protein
BiP: Binding immunoglobulin protein
BDFACS: BD Biosciences fluorescence activated cell sorting
BRAF: B-Raf proto-oncogene serine/threonine kinase
CD47: Integrin associated protein
CICD: Caspase-independent cell death
cPARP: cleaved poly ADP ribose polymerase
CRT: calreticulin
CsA: cyclosporin A
ER: endoplasmic reticulum
FACS: fluorescence activated cell sorting
FCCP: Carbonyl cyanide-4-(trifluoromethoxy)phenylhydrazone
GAPDH: Glyceraldehyde 3-phosphate dehydrogenase
GFP: Green fluorescence protein
HCT116: Human colorectal carcinoma cell line
HeLa: Human cervical adenocarcinoma cell line
HR: hazard ratio
ICD: immunogenic cell death
IP: immunoprecipitation
mAb: monocloncal antibody
MB: megabase
MDAMB361: human breast adenocarcinoma cell line
MFI: mean fluorescence intensity
mito-GFP-CaMP33: mitochondrial genetically encoded calcium indicator 3, created from a fusion of green fluorescent protein, calmodulin, and M13, a peptide sequence from myosin light chain kinase
MOMP: mitochondia outer membrane pore
mPTP: mitochondria permeability transition pore
NIR: Near-Infrared
NRF: necrosis related factor
PARP: poly ADP ribose polymerase
PI3K: Phosphoinositide 3-kinase
pLNeo: lenti-viral plasmid expressing Neomycin R gene
qRT-PCR: Real-Time Quantitative Reverse Transcription PCR
RCC4: Human clear cell renal cell carcinoma cell line
RFU: Relative fluorescent unit
shCont: short hairpin RNA targeting Control
shCRT: short hairpin RNA targeting Calreticulin
shRNA: short hairpin RNA
SKOV1: Human ovarian carcinoma cell line
SS: Staurosporin
TC: tissue culture
TCGA: The cancer genome atlas
TSC2: Tuberous Sclerosis Complex 2 gene
UVC: Ultraviolet C
