# Supplementary figures and images for: Calreticulin is a Critical Cell Survival Factor in Malignant Neoplasms
Source: PLoS Biol. 2019 Sep 30;17(9):e3000402. doi: 10.1371/journal.pbio.3000402 (PMC6768457; doi:10.1371/journal.pbio.3000402)

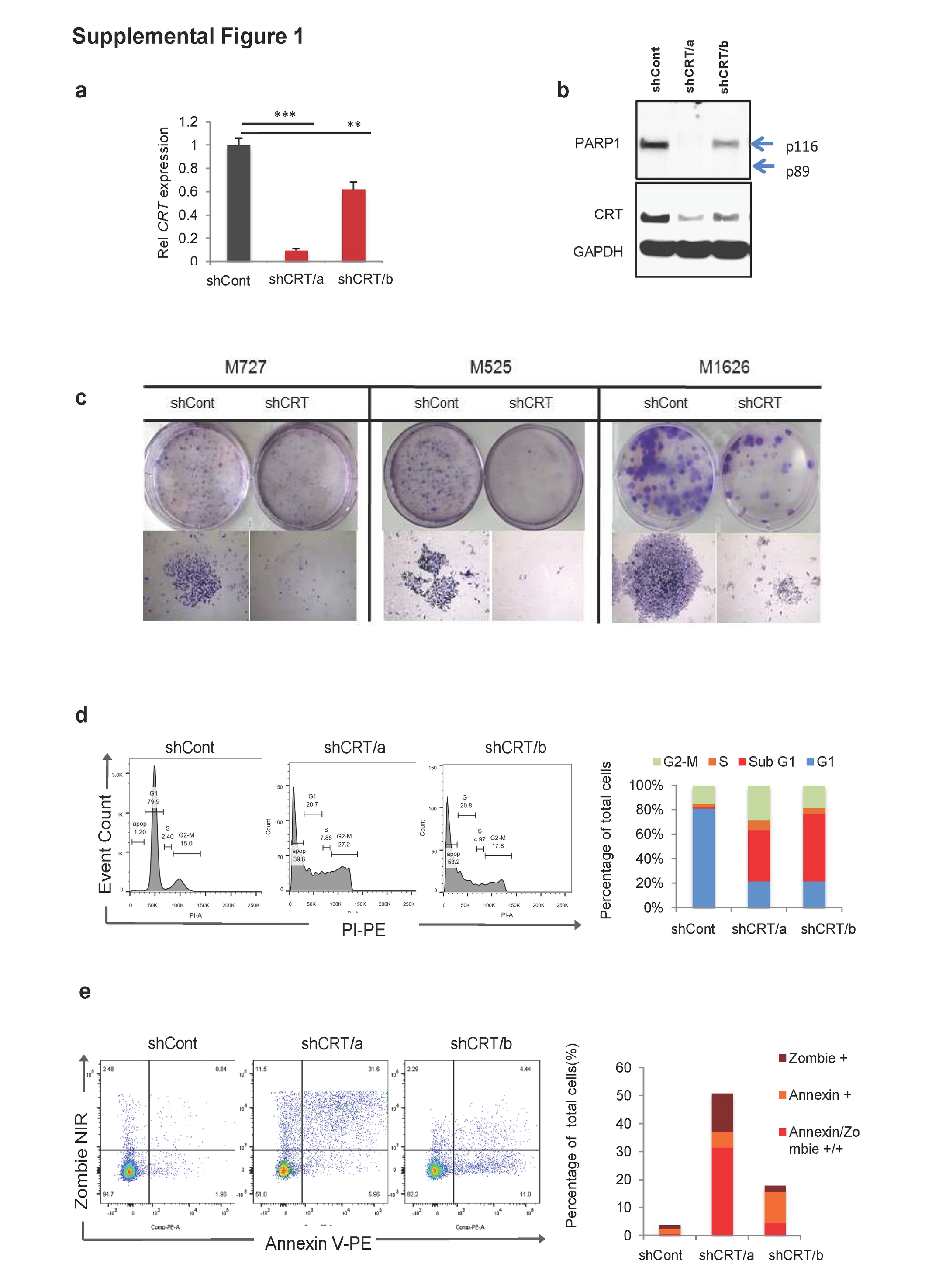

Supplement: S1 Fig — (A) Quantification of CRT mRNA levels in shCRT/a, shCRT/b, and shCont transduced cells using qRT-PCR and CRT-specific primers. (B) Analysis of CRT and PARP 1 protein levels in shCRT/a, shCRT/b, and shCont transduced cells using Western blot. (C) Representative images of the colony numbers and sizes formed by the shCont or shCRT-transduced melanoma cells (M727, M525, and M1626) following their fixation and crystal violet staining. (D) Cell cycle analysis of shCRT/a, shCRT/b, and shCont transduced cells after they were stained with PI and analyzed using flow cytometry. The number of cells in various stages of the cell cycle was quantified by measuring the area under the peaks (sub-G1, G1, S, G2−M phases). (E) Analysis and quantification of cell death phenotype in shCRT/a, shCRT/b, and shCont transduced cells using Zombie/Annexin V stain and flow cytometry. Underlying source data can be found in S1 Data. CRT, calreticulin; PARP, poly ADP ribose polymerase; PI, propidium iodide; qRT-PCR, Real-Time Quantitative Reverse Transcription PCR; shCont, short hairpin RNA targeting Control; shCRT, short hairpin RNA targeting Calreticulin; shRNA, short hairpin RNA. (TIF) [file pbio.3000402.s001.tif]

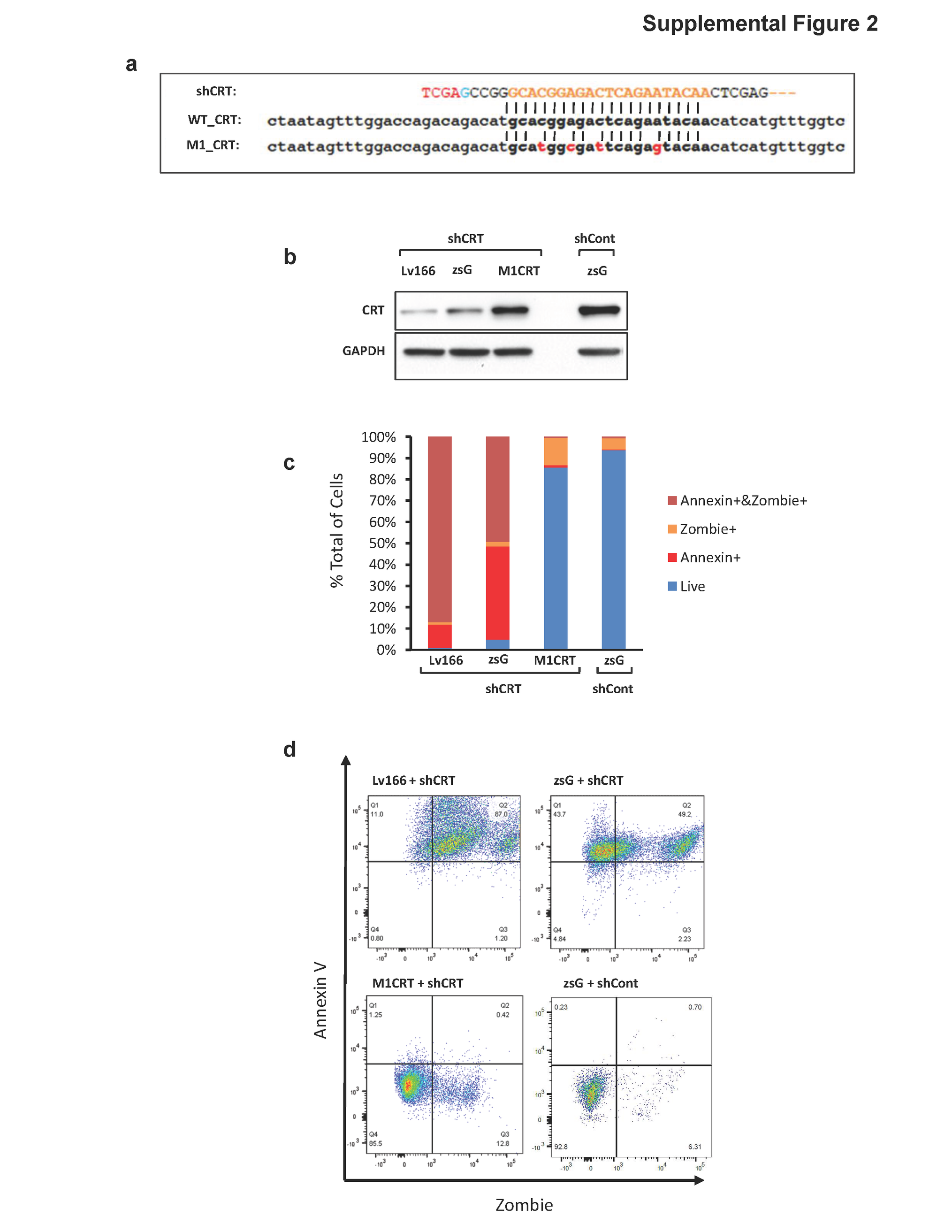

Supplement: S2 Fig — (A) Sequence alignment of shCRT, wild-type CRT, and shCRT insensitive M1, CRT mutant. (B) Analysis of CRT protein levels in the target cells transduced with the combination of indicated plasmids and assayed using WB and CRT-specific antibodies. (C) Quantification of cell viability using Annexin/Zombie fluorescent assay following their transduction with the combination of indicated plasmids. (D) Representative FACS plots of the viability stains. Underlying source data can be found in S1 Data. CRT, calreticulin; FACS, fluorescence activated cell sorting; shCRT, short hairpin RNA targeting Calreticulin; WB, Western blot. (TIF) [file pbio.3000402.s002.tif]

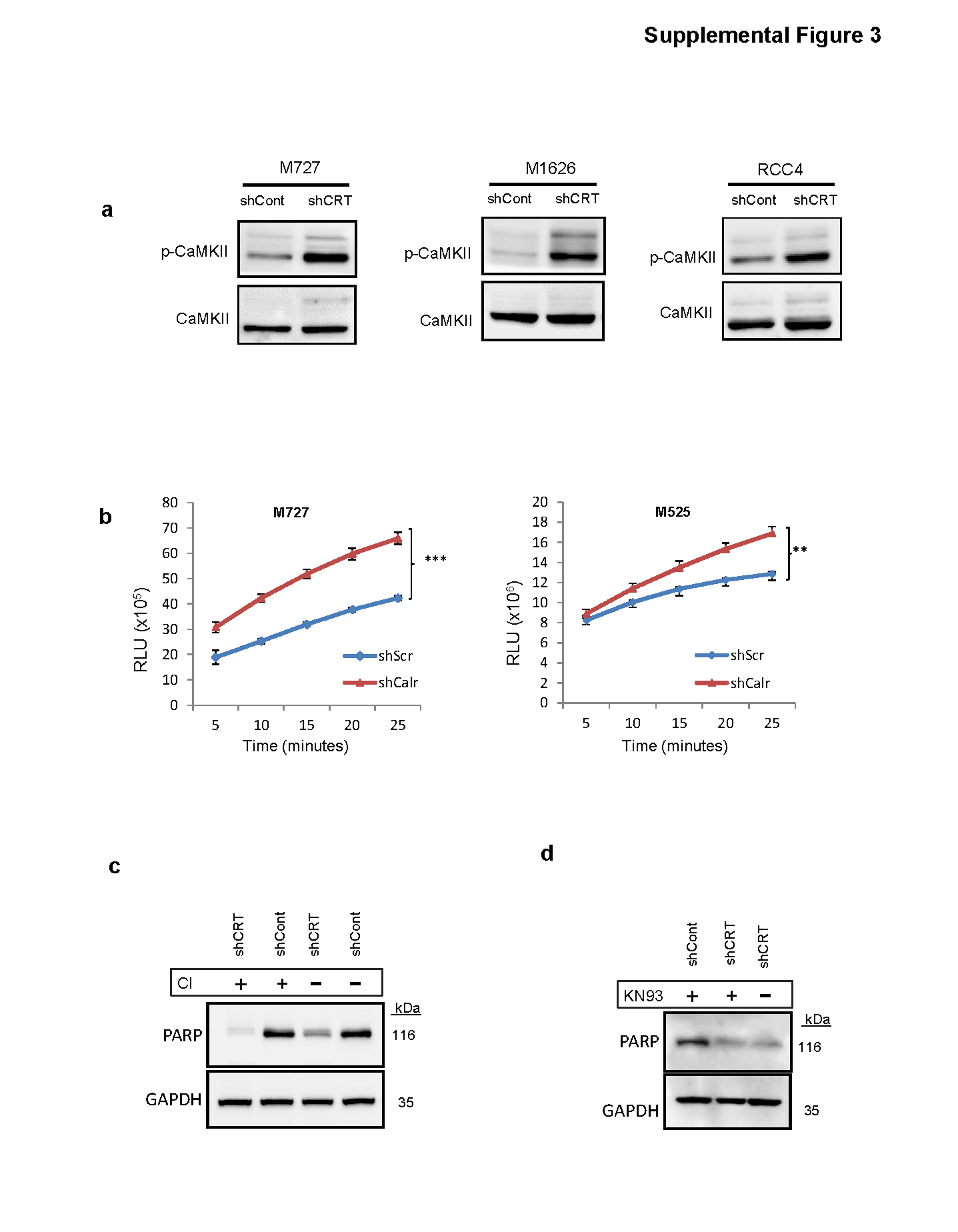

Supplement: S3 Fig — (A) Protein level analysis of phospho- and pan- CaMKII using Western blot in the indicated solid tumor cells following their transduction with shCont or shCRT. (B) Quantification of the Calpain activity in shCont- or shCRT-transduced cells at indicated time points using Calpain-Glo assay. Unpaired Student t test was used to calculate P-values (**P < 0.005, ***P < 0.0005). All error bars indicate mean ± SD. Analysis of full-length PARP protein levels using Western blot following incubation of the indicated cells with CI (C) or CamKII inhibitor, KN95 (D). Underlying source data can be found in S1 Data excel table. CI, Calpain inhibitor; PARP, poly ADP ribose polymerase; shCont, short hairpin RNA targeting Control; shCRT, short hairpin RNA targeting Calreticulin. (TIF) [file pbio.3000402.s003.tif]

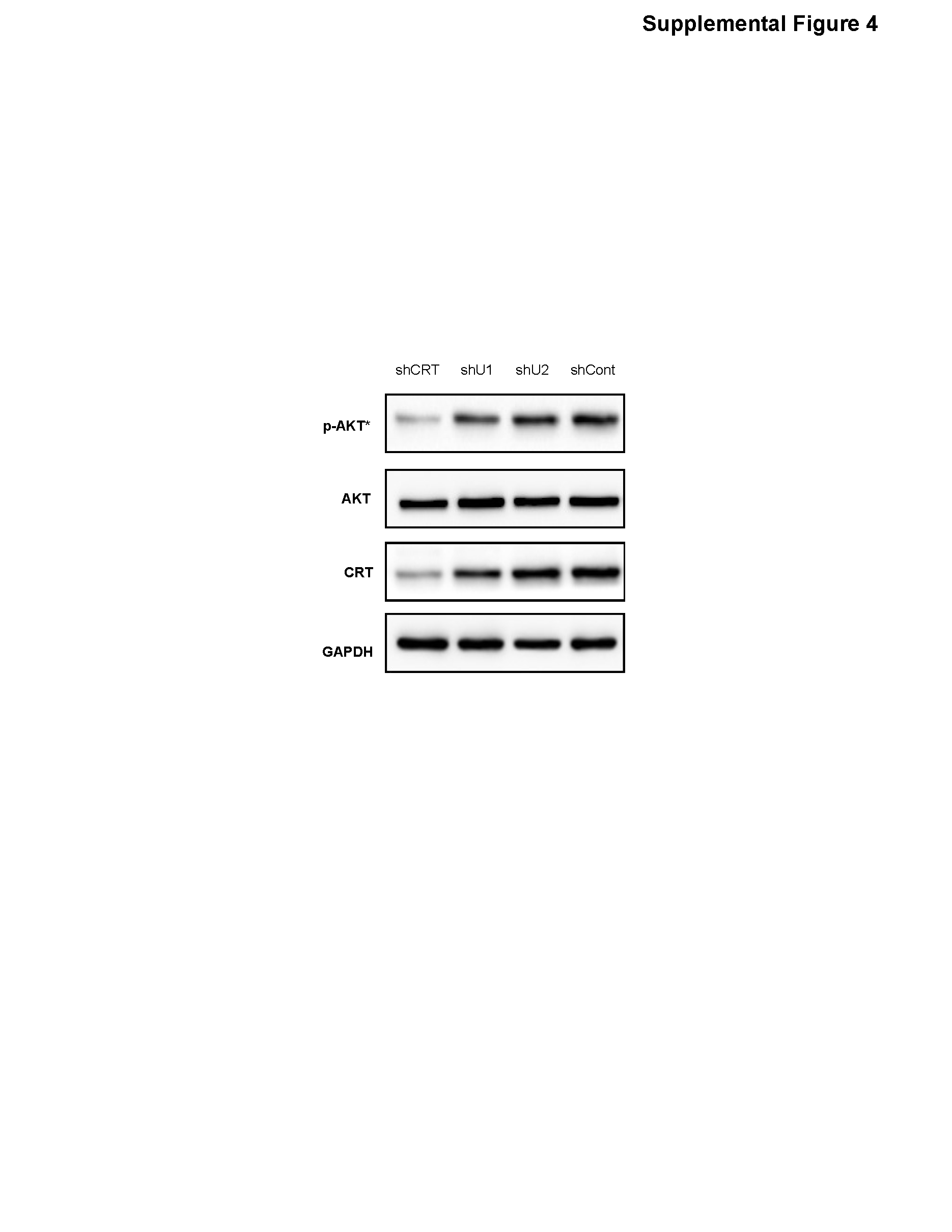

Supplement: S4 Fig — Analysis of AKT-PSer473, total AKT, and CRT protein levels using WB and respective antibodies following transduction of target cells with shCRT, CRT-nontargeting shRNAs (shU1 and shU2), and shCont. AKT, Protein kinase B; CRT, calreticulin; shCont, short hairpin RNA targeting Control; shCRT, short hairpin RNA targeting Calreticulin; shRNA, short hairpin RNA; WB, Western blot. (TIF) [file pbio.3000402.s004.tif]
