# Supplementary material for: Calreticulin is a Critical Cell Survival Factor in Malignant Neoplasms
Source: PLoS Biol. 2019 Sep 30;17(9):e3000402. doi: 10.1371/journal.pbio.3000402 (PMC6768457; doi:10.1371/journal.pbio.3000402)
Supplement: S1 Table — (PDF) [file pbio.3000402.s006.pdf]

qRT-PCR primer sequences

Gene	Gene symbol	Sequence (5’ à 3’)
Calreticulin (NM_004343.3)	CRT	Forward
		AGGATGATGAGTTTACACAC
		Reverse
		TCATCGATCTTGGCCCGCTC
BCL2 binding component 3	PUMA	Forward
(NM_001127240.2)		GACCTCAACGCACAGTACGAG
		Reverse
		AGGAGTCCCATGATGAGATTGT
BCL2 associated X, apoptosis regulator	BAX	Forward
(NM_001291428.1)		CCCGAGAGGTCTTTTTCCGAG
		Reverse
		CCAGCCCATGATGGTTCTGAT
18S ribosomal RNA	18S rRNA	Forward
		GCCCGAAGCGTTTACTTTGA
		Reverse
		TCCATTATTCCTAGCTGCGGT
